# Esophageal Epithelial-Derived IL-33 Is Upregulated in Patients with Heartburn

**DOI:** 10.1371/journal.pone.0154234

**Published:** 2016-04-25

**Authors:** Hiroo Sei, Tadayuki Oshima, Jing Shan, Liping Wu, Takahisa Yamasaki, Takuya Okugawa, Takashi Kondo, Toshihiko Tomita, Hirokazu Fukui, Jiro Watari, Hiroto Miwa

**Affiliations:** 1 Division of Gastroenterology, Department of Internal Medicine, Hyogo College of Medicine, Nishinomiya, Japan; 2 Depertment of Gastroenterology, The Third People’s Hospital of Chengdu, Chengdu, China; Shiga University of Medical science, JAPAN

## Abstract

**Background:**

Interleukin-33 (IL-33) is a tissue-derived cytokine that is constitutively expressed in epithelial cells of tissues exposed to the environment and plays a role in sensing damage caused by inflammatory diseases. IL-33 acts as both a traditional cytokine and as a chromatin-associated nuclear factor in both innate and adaptive immunity. We recently showed that IL-33 in esophageal mucosa is upregulated in reflux esophagitis. However, IL-33 expression in patients with heartburn without mucosal injury and its relationship with intercellular space (ICS) have never been examined. We therefore examined the expression of cytokines and ICS in patients with heartburn.

**Methods:**

The expression of IL-33 in the middle and distal esophageal mucosa of patients with heartburn without mucosal break and control samples was examined using real-time RT-PCR and immunofluorescence. The mRNA expression of IL-6, IL-8, MCP-1, and RANTES, and ICS was also analyzed.

**Results:**

IL-33 expression and the mean ICS were significantly increased in the mucosa of patients with heartburn compared to that of the control. IL-33 and ICS were not different between the patients who were taking a PPI and those who were not. The upregulated IL-33 expression in the heartburn group was located in the nuclei of the basal cell layer. Although IL-6, IL-8, MCP-1 and RANTES levels were not different between control and patients with heartburn samples, IL-33 mRNA levels were still significantly correlated with IL-6, IL-8, or MCP-1 mRNA levels.

**Conclusion:**

Nuclear IL-33 is upregulated in patients with heartburn. Upregulated IL-33 in heartburn patients is related to the symptoms.

## Introduction

Gastroesophageal reflux disease (GERD) is one of the most common gastrointestinal diseases and its incidence is increasing [1]. Since acid is the major cause of GERD symptoms, proton pump inhibitor (PPI) therapy relieves the symptoms. However, a subset of GERD patients do not attain adequate symptom relief with a PPI [2, 3]. Since esophageal inflammation occurs prior to macroscopic or even microscopic signs of mucosal injury in GERD, a new view has been proposed of the cause of this disease. In this new model it is suggested that gastroesophageal reflux (GER) causes esophagitis through a cytokine-mediated mechanism rather than through caustic acid-induced direct mucosal injury and that cytokines are upregulated in erosive esophagitis [4]. Acid suppressive therapy is less effective for non-erosive reflux disease (NERD) patients than it is for erosive reflux esophagitis (RE) patients [5, 6] and esophageal visceral hypersensitivity and impaired mucosal integrity have been proposed as possible mechanisms of disease in NERD patient [7–9].

Interleukin-33 (IL-33) was originally described as a nuclear factor that is preferentially expressed in human high endothelial venules [10]. IL-33 is a member of the IL-1 family that includes cytokines such as IL-1β and IL-18 [11]. Exogenous IL-33 activates the release of T helper 2 (Th2) cytokines such as IL-4, IL-5 and IL-13 through its interaction with the IL-1-receptor related protein, ST2 [11]. IL-33 also binds NF-kB directly as a nuclear factor, thereby regulating gene transcription or activating the production of inflammatory cytokines [12–14]. We recently reported that IL-33 is upregulated in the erosive mucosa of RE patients and that epithelial-derived nuclear IL-33 enhances the release of cytokines from epithelial cells and aggravates inflammation in the pathogenesis of RE [14, 15]. Upregulated IL-33 was correlated with the expression of IL-8 and IL-6. However, IL-8 and IL-6 production in NERD is controversial and there is no report that shows the expression of IL-33 in patients with heartburn without mucosal break. How IL-33 is related to GERD symptoms is also not clear.

Dilated intercellular spaces (DIS) in the esophageal epithelial cell layers of RE and NERD patients have been proposed as a marker of GER, and have been extensively studied [16–18]. Although DIS are not always related to the symptoms of GER, acid or other irritants do induce DIS. Acid perfusion in the esophagus of healthy volunteers causes DIS in initially normal epithelium [19]. Interestingly DIS was found in basal cell layers but not in granular cell layers and was found without inflammation.

In this study, to explore the production of IL-33 and other inflammatory cytokines in patients with heartburn without mucosal break, we examined mucosal biopsies and evaluated the expression pattern of cytokines including IL-33, IL-6, IL-8, and monocyte chemotactic protein-1 (MCP-1) in patients with heartburn, using DIS as a marker of GER.

## Material and Methods

### Human endoscopic biopsies

Twenty eight patients with heartburn who did not have any lesion in the upper gastrointestinal tract on endoscopy and 25 controls were recruited at Hyogo College of Medicine between 2012 and 2015. Inclusion criteria for the heartburn group were recurrent typical reflux symptoms and symptom duration of more than two months. Exclusion criteria for all participants were as follows: eosinophilic esophagitis, proton pump inhibitor-responsive esophageal eosinophilic infiltration [20], previous history of erosive reflux esophagitis, intake of nonsteroidal anti-inflammatory drugs [21], corticosteroids, anti-allergic drugs, or other immunosuppressive drugs in the preceding two months; and allergy or inflammatory bowel diseases. Endoscopic biopsy samples were taken from unaffected mucosa of the middle (approximately 30 cm from the incisor tooth) and distal (above the esophagogastric junction) esophagus of patients with heartburn and control groups. The control group was subjects who received endoscopy without symptoms and medications, and who were with normal endoscopy. One biopsy sample was immediately stored in RNAlater (Qiagen, Hilden, Germany), and was maintained at -20°C until the measurement of messenger RNA (mRNA). Another biopsy sample for analysis using immunofluorescence and hematoxylin and eosin (HE) staining was fixed with 10% neutral formalin and embedded in paraffin.

Patient anonymity was preserved. This study was performed in accordance with the Declaration of Helsinki and was approved by the Ethics Committee/Institutional Review Board of Hyogo College of Medicine, Japan (no. 174). The subjects gave written informed consent.

### Reverse transcription quantitative PCR

Total mRNA was extracted using the Trizol reagent according to the manufacturer’s instructions (Invitrogen Life Technologies, Carlsbad, CA). Complementary DNA was synthesized using a high capacity complementary DNA reverse transcription (RT) kit (Applied Biosystems, Foster City, CA). Quantitative PCR (qPCR) was performed using a PCR master mix and a 7900HT fast real-time PCR system (Applied Biosystems).

The TaqMan probe and primers for IL-33 (accession no. Hs00369211; Applied Biosystems), IL-8 (Hs00174103), IL-6 (Hs00985639), MCP-1 (Hs00234140), and regulated on activation, normal T cell expressed and secreted (RANTES) (Hs00982282) were assay-on demand gene expression products. The GAPDH gene (Hs02758991) was used as the endogenous control. The thermal cycler conditions were as follows: 2 min at 50°C, 10 min at 95°C, followed by 40 cycles of 15 s at 95°C for denaturing and 1 min at 60°C for annealing/extension. All procedures were repeated in triplicate. Amplification data were analyzed with Sequence Detection System version 2.2 (Applied Biosystems). The ΔΔCT method recommended by the manufacturer was used to compare the relative expression levels.

### Measurement of histological parameters

All mucosal biopsy specimens were stained with HE. The diameters of intercellular spaces (ICSs) between squamous epithelial cells in the middle and distal esophagus was measured using computer-assisted morphometry (×800 magnification) (cellSens; OLYMPUS, Tokyo, Japan) [22]. The histological parameters were evaluated as previously described with minor modifications [23–25]. In brief, basal cell thickness (normal values: < 15%) and length of papillae (normal values: < 66%) were recorded as a percentage of the total epithelial thickness. When lesions were not homogeneously distributed in a given sample, the most severe change was considered. The presence of intraepithelial infiltration of eosinophils (normal values: 0), and neutrophils (normal values: 1 or less) was recorded. The mean of the most infiltrated three high power fields (40 x) were calculated. The presence of patients with the increased number of eosinophils and neutrophils were assessed.

### Immunofluorescence staining

Paraffin-embedded sections (4 μm thick) of the esophagus were deparaffinized, heated in citrate buffer (pH 6.0) for epitope retrieval, and then blocked with phosphate-buffered saline containing 5.0% bovine serum albumin. The sections were incubated with primary rabbit anti-IL-33 (MBL, Nagoya, Japan) at 4°C overnight. Subsequently, the sections were reacted with Cy3-conjugated goat anti-rabbit IgG (Bethyl Laboratories, Montgomery, TX) at room temperature and then nuclei were counterstained with 4’, 6-diamidino-2-phenylindole (DAPI). The sections were examined under a microscope (Zeiss LSM 780; Carl Zeiss, Thornwood, NY). Computer software (ZEN 2012; Carl Zeiss) was used for image processing.

### Statistical analysis

Two-tailed Mann-Whitney U-test, Kruskal-Wallis test or unpaired t-test was performed where appropriate. Fisher's exact test with Bonferroni correction for multiple testing was performed for histological evaluations. ICS diameter is presented as the mean ± standard deviation. Nonparametric correlation analysis was performed using Spearman’s rank correlation coefficient. All tests were applied two-sided with a significance level of *P* < 0.05. GraphPad Prism6 (GraphPad Software, Inc. La Jolla, CA) was used for statistical analysis.

## Results

### Intercellular space diameter of the esophagus in patients with heartburn and control samples

A total of 53 subjects were examined; 25 controls and 28 patients with heartburn. Age and sex were not different between the controls and the heartburn patients (62.0 ± 17.7 vs. 67.3 ± 9.4 years, and 9 vs. 11 males, respectively). Of the 28 heartburn patients studied, 20 (71%) were taking a PPI (2 subject was taking omeprazole, 4 lansoprazole, 7 rabeprazole, and 7 esomeprazole) at the time of examination.

**Fig 1:**
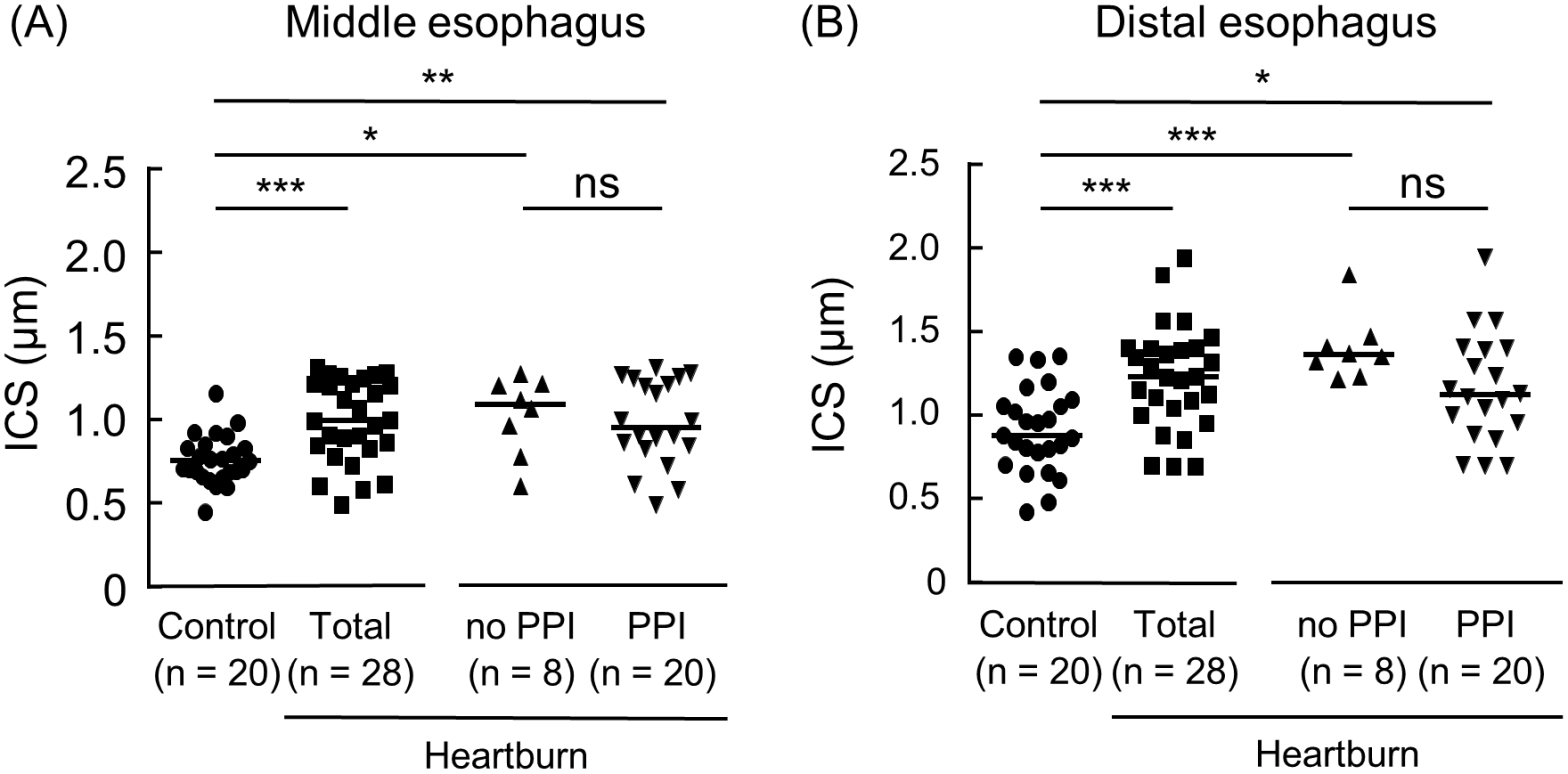
Intercellular space (ICS) diameters of the esophagus in patients with heartburn patients and controls. Middle and distal esophageal biopsy specimens were stained with hematoxylin and eosin. Intercellular space diameters of the middle (A) and distal (B) esophagus at the basal layers of squamous epithelial cells were then measured in control samples and heartburn patients (Total). The heartburn patients (Total) consist of the subjects who were not taking a PPI (no PPI) and who were (PPI). The horizontal line in each sample indicates the median value. *P < 0.05, **P < 0.01, ***P < 0.001 vs. Control; ns, not significant vs. no PPI.

The average ICS diameter of the esophagus was calculated for each sample. The ICS diameters of both the middle and distal esophagus were significantly wider in the heartburn group than those in the control group (*P* < 0.001) (Fig 1). The ICS diameters of the middle and distal esophagus of the heartburn patients who were and were not taking a PPI were also significantly wider compared to those of the control group (Fig 1). Basal cell hyperplasia of the total heartburn patients or the heartburn patients who were taking a PPI was significantly more frequent than the controls. However, elongation of papilla, neutrophil or eosinophil infiltrations were not different between the controls and the heartburn patients (Table 1). All histological parameters were not different between the heartburn patients who were taking a PPI and those who were not.

**Table 1:** Histological characteristics in patients and controls.

	Control (n = 13)	Heartburn					
		Total (n = 18)	*P*^#^	no PPI (n = 7)	*P*^#^	PPI (n = 11)	*P*^#^
Basal cell hyperplasia	5	15	< 0.05	5	ns	10	< 0.05
Elongation of the papilla	4	9	ns	4	ns	5	ns
Neutrophil infiltration	0	4	ns	1	ns	3	ns
Eosinophil infiltration	0	1	ns	0	ns	1	ns

The number of subjects with each positive histological parameter is shown. PPI, proton pump inhibitor. The heartburn patients (Total) consist of the subjects who were not taking a PPI (no PPI) and who were (PPI). *P*^#^ vs. Control (Fisher’s exact test with Bonferroni correction); ns, not significant vs. Control.

### IL-33 expression in the esophageal epithelial layer of heartburn patients and controls

**Fig 2:**
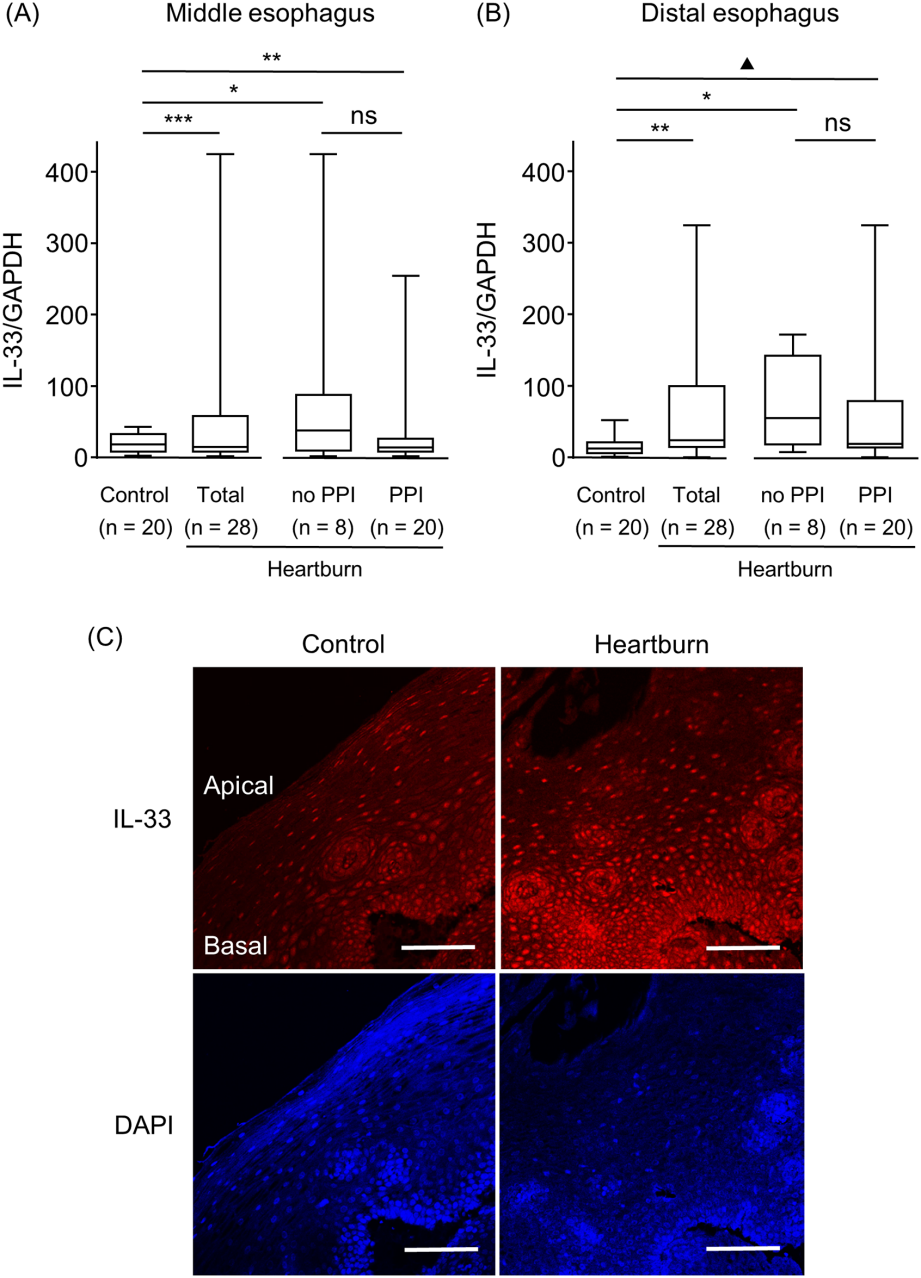
IL-33 expression in esophageal epithelial layers of patients with heartburn and controls. IL-33 mRNA expression was analyzed using qRT-PCR and was compared at the middle (A) and distal (B) esophagus of control samples, and the heartburn patients (Total), the subjects who were not taking a PPI (no PPI) and who were (PPI). (C) Representative immunofluorescence staining of IL-33 in the apical and basal layers of the distal esophagus of a control sample and a heartburn patient with taking a PPI is shown. DAPI was used for nuclear staining. *P < 0.05, **P < 0.01, ***P < 0.001, ^**▲**^*P* = 0.08 vs. Control; ns, not significant vs. no PPI. Bar = 100 μm.

IL-33 mRNA expression in the middle and distal esophagus was compared between the control and the heartburn groups using qRT-PCR. The IL-33 mRNA level in the distal but not in the middle esophagus was significantly increased in the heartburn group compared to the control group (*P <* 0.01) (Fig 2). The IL-33 mRNA level of the distal esophagus of the patients who were not taking a PPI was significantly higher compared to that of the control group (*P* <0.05). The IL-33 mRNA level of the distal esophagus of the patients who were taking a PPI was also tend to be higher compared to that of the control group (*P* = 0.08). The IL-33 mRNA levels were not different between the heartburn patients who were taking a PPI and those who were not. Representative immunofluorescence staining of the distal esophagus showed that the upregulated IL-33 expression in the heartburn group with taking a PPI was located mainly in the nuclei of the basal cell layer (Fig 2). The level of IL-8, IL-6, MCP-1 or RANTES mRNA did not differ between control and heartburn groups (Table 2).

**Table 2:** Relative levels of cytokine mRNAs.

	Middle esophagus							Distal esophagus						
	Control (95%CI)	Heartburn						Control (95%CI)	Heartburn					
		Total (95%CI)	*P*^#^	no PPI (95%CI)	*P*^##^	PPI (95%CI)	*P*^##^		Total (95%CI)	*P*^#^	no PPI (95%CI)	*P*^##^	PPI (95%CI)	*P*^##^
IL-6	1.00 (0.66–1.61)	0.90 (0.57–1.53)	0.77	1.47 (0.38–14.10)	0.94	0.76 (0.51–1.07)	0.99	1.00 (0.68–2.63)	1.10 (0.82–2.17)	0.72	1.73 (0.62–10.90)	0.99	1.04 (0.68–2.16)	0.99
IL-8	1.00 (0.62–1.94)	2.06 (0.91–4.47)	0.09	4.47 (0.63–334.10)	0.06	1.51 (0.75–4.32)	0.99	1.00 (0.41–1.92)	1.23 (0.68–2.91)	0.35	1.23 (0.09–86.49)	0.99	1.27 (0.35–4.39)	0.69
MCP-1	1.00 (0.61–1.46)	0.85 (0.58–1.15)	0.42	1.00 (0.15–5.10)	0.99	0.78 (0.58–1.151)	0.87	1.00 (0.63–1.18)	0.99 (0.59–1.53)	0.77	0.98 (0.27–1.96)	0.99	0.99 (0.59–1.55)	0.99
RANTES	1.00 (0.70–1.12)	0.91 (0.70–1.38)	0.99	1.18 (0.28–4.80)	0.83	0.77 (0.53–1.38)	0.99	1.00 (0.48–2.18)	0.61 (0.37–0.85)	0.28	0.69 (0.37–2.79)	0.99	0.55 (0.29–0.85)	0.67

CI, confidence interval; MCP-1, monocyte chemotactic protein-1; RANTES, regulated on activation, normal T cell expressed and secreted. *P*^#^ vs. Control (Mann-Whitney U-test), *P*^##^ vs. Control (Kruskal-Wallis test)

**Fig 3:**
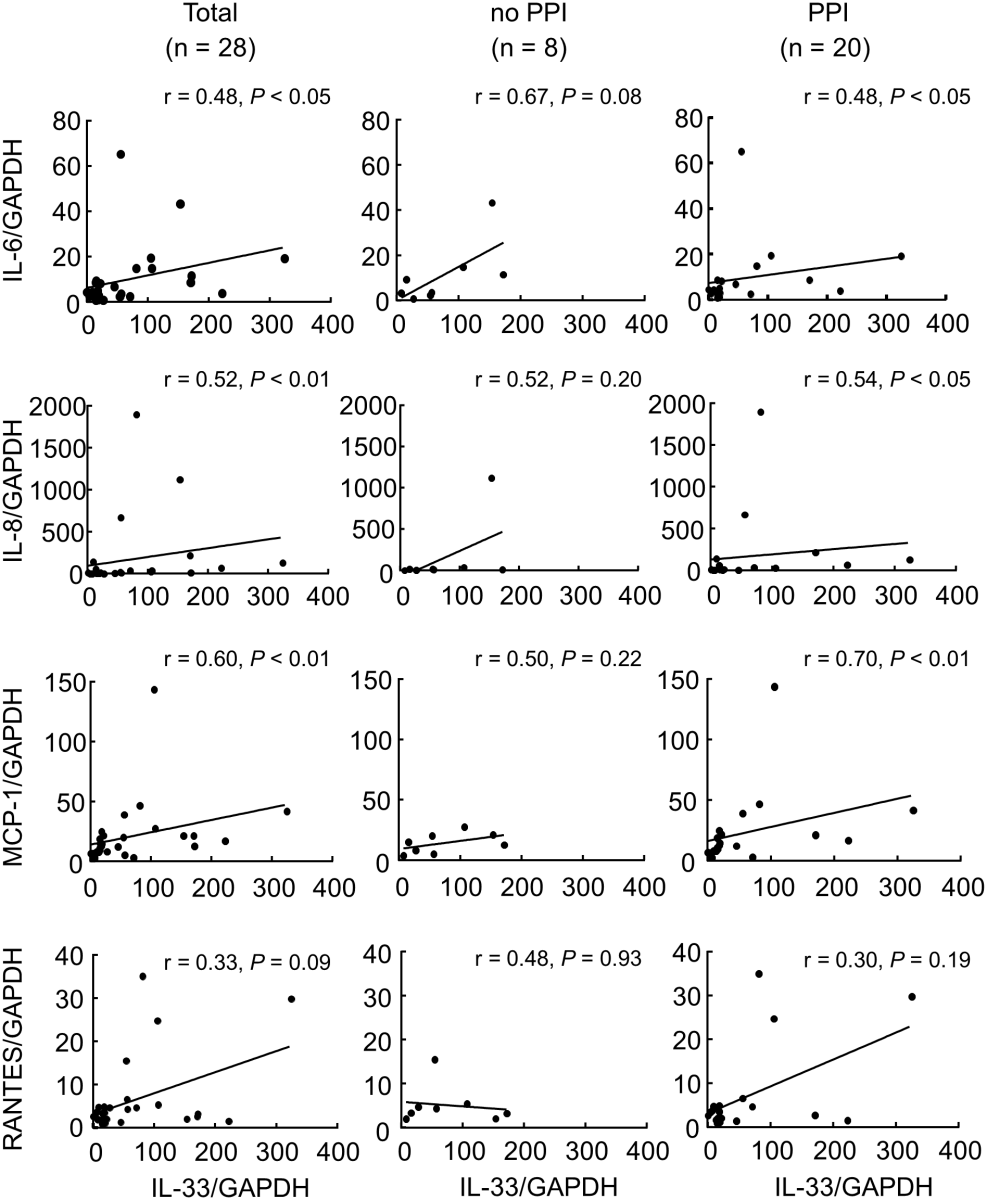
Analysis of the correlation of IL-33 and other inflammatory cytokines. Correlation of IL-33 mRNA levels and the mRNA levels of (A) IL-6, (B) IL-8, (C) MCP-1 and (D) RANTES in biopsy specimens from the distal esophagus of patients with heartburn, and patients who were not taking a PPI (no PPI) and who were (PPI) was performed by calculation of Spearman’s rank correlation coefficient.

The level of IL-33 mRNA was significantly correlated with ICS diameter (r = 0.33, *P* < 0.05). The correlation between the IL-33 mRNA level and the mRNA levels of IL-6, IL-8, MCP-1 and RANTES in patients with heartburn biopsy distal esophagus specimens was also analyzed. The level of IL-33 mRNA was significantly correlated with the mRNA level of IL-8, IL-6, and MCP-1 (Fig 3). The level of IL-33 mRNA was not correlated with the mRNA level of RANTES (Fig 3). The IL-33 mRNA of the patients who were taking a PPI was also significantly correlated with the mRNA level of these cytokines.

## Discussion

Esophageal epithelial layers maintain a barrier against caustic chemical injury [26–29]. However, recent studies have shown that epithelial cells can initiate inflammation by producing various inflammatory cytokines including IL-8 [4]. Here, we demonstrated that IL-33 but not IL-8 or MCP-1 was upregulated in heartburn patients and that this upregulated IL-33 expression was localized in the nuclei of the basal side of the epithelial layers. IL-33 was originally reported as a nuclear factor that can sense damage caused by various inflammatory diseases [30, 31]. We recently reported that IL-33 is upregulated in the nuclei of esophageal epithelial cell layers in RE patients and that this IL-33 enhances the release of IL-8 and IL-6 followed by aggravation of the inflammatory status of the esophagus [14]. Although growing evidence suggests that NERD is not a mild form of RE and that these entities are two distinct categories of GERD, the data presented here indicated that upregulation of IL-33 was seen in both heartburn patients without mucosal break and RE, and seemed to be related to the existence of GER and to GERD symptoms.

The IL-33 level was found to be related to the aggravation of inflammatory cytokine production including IL-8 and IL-6 in our previous study of RE patients [14]. IL-33 might therefore be a key cytokine for the development of RE and IL-33 production may precede the upregulation of inflammatory cytokines. Based on previous reports that indicated an increase in inflammatory cytokines in NERD, we speculated that these cytokines would also be upregulated in the heartburn patients of the present study. However, the mRNA levels of IL-8, IL-6, MCP-1 and RANTES were not upregulated in heartburn patients regardless the use of PPI versus controls in this study, even though the IL-33 mRNA level in heartburn patients still correlated with IL-8 and IL-6 levels. These results indicated that IL-33 levels might be more sensitive to GER and may change in a more consistent manner with GER. Since IL-33 is a factor in the enhancement of inflammatory cytokine release from esophageal epithelial cell layers, other additional triggers might be needed for the upregulation of IL-8, MCP-1 and IL-6. Thus the release of these inflammatory cytokines may also be related to the development of macroscopic mucosal injury such as erosion.

The existence of GER is often diagnosed by empirical PPI treatment, and endoscopy can confirm the existence of erosion. A PPI test is recommended by the guidelines and is beneficial in that it can reduce the number of expensive and redundant endoscopy examinations. However, a systematic review and a recent report indicated that the specificity of the PPI test is not very high [32, 33]. In NERD patients, conventional endoscopy just shows no evidence of mucosal damage and cannot detect the existence of GER. Furthermore, an ambulatory impedance-pH monitoring test is uncomfortable, inconvenient, and time consuming for patients. Therefore, establishment of a reliable way to indicate the existence of GER in heartburn patients is essential.

Elongated papillae, basal hyperplasia, and DIS are evaluated as markers of GER. As acid perfusion in the esophagus induces DIS without symptoms in healthy subjects and DIS can be reversed by PPI treatment, DIS can be used as a marker of GER [19, 28, 34]. Basal cell hyperplasia and elongation of papillae were also reported as candidate markers of NERD [35]. However, how these markers are correlated to DIS still needs further investigation because DIS and basal hyperplasia were reported to be related to IL-1β or IL-8 levels, which were not upregulated in the heartburn patients in this study. Furthermore, DIS evaluation is needed to minimize interobserver differences [36]. Therefore, these indices are not complete and new markers that can indicate GER and GERD symptoms will be useful.

ICS diameter and IL-33 levels were correlated in this study, indicating that IL-33 is related to GER and GERD symptoms. Interestingly, the production of IL-8 or other inflammatory cytokines did not consistently correlate with heartburn patients regardless the use of PPI. Although IL-8 mRNA has been reported to be upregulated in NERD [37–39], a recent report showed no increase in IL-8 or MCP-1 in NERD patients [40]. That report still showed that inflammatory cytokines including IL-8 and MCP-1 were upregulated in RE. An increase in IL-8 and MCP-1 has been consistently reported in RE and is correlated with histological severity [39, 41, 42]. The data presented here and the data that we previously reported were in agreement with the latter report. These differences in RE and patients with heartburn without mucosal break might be related to the degree of inflammatory cell infiltration and thus the upregulated IL-8 mRNA level might be affected not only by epithelial cells but also by these inflammatory cells. In other words, these differences may depend not only on epithelial modification but also on inflammatory cell infiltration. Indeed, a recent report also indicated that basal hyperplasia, DIS and papillary elongation were seen even in NERD but that immune cell infiltration was not observed in NERD [40, 43].

Another explanation of the inconsistencies in inflammatory cytokine expression might be related to the heterogeneities of the studied patients and to the use of acid suppressive agents including a PPI. Two thirds of the heartburn patients were PPI refractory in this study and thus the use of PPI might modulate the level of IL-8 or other cytokines except for IL-33 [37]. These data indicated that IL-33 might be independent of PPI use and may thus be a good biomarker for GER and its symptoms without being affected by PPI.

Regarding the limitations of this study, first, we included patients with heartburn but did not examine exact GER with a 24-h impedance-pH monitoring test. However, we did evaluate ICS as a marker of GER and the IL-33 level was correlated with ICS, indicating that GER was related to the upregulation of IL-33 and heartburn in this study. Second, we included patients who took acid suppressive agents including a PPI. It has been reported that exposure to acidic bile salt medium induces IL-8 release from esophageal squamous cells and that omeprazole inhibits IL-8 expression through effects on nuclear factor-κB and activator protein-1 [44]. However, we focused on heartburn in this study, and based on the data of DIS and IL-33 in heartburn patients, even in those patients with PPI treatment, it is possible that IL-33 levels might be related to the development and persistence of GERD symptoms regardless of the use of PPI. However, further examinations are needed to clarify the function of IL-33 and to elucidate how IL-33 is involved in the development of heartburn symptom. Third, the number of the heartburn patients who were not taking PPI was still small and the data from them may not be conclusive.

In summary, we showed that IL-33 levels were upregulated in patients with heartburn. IL-33 levels were not different between patients who were taking a PPI and those who were not. Further studies are needed to explore the mechanisms by which IL-33 is regulated in patients with heartburn and how IL-33 levels are related to the generation of heartburn symptoms.
