# Correction: Esophageal Epithelial-Derived IL-33 Is Upregulated in Patients with Heartburn

**DOI:** 10.1371/journal.pone.0158932

**Published:** 2016-07-06

**Authors:** Hiroo Sei, Tadayuki Oshima, Jing Shan, Liping Wu, Takahisa Yamasaki, Takuya Okugawa, Takashi Kondo, Toshihiko Tomita, Hirokazu Fukui, Jiro Watari, Hiroto Miwa

There is a sizing error in the vertical axis values of Fig 1B. There is an error in the statistical significances of Fig 2A. Values “*”, “**”, and “***” should read “ns”, “ns”, and “ns”. There is an error in the last sentence of the caption for Fig 2. “***P < 0.001” should be erased. “DAPI was used for nuclear staining. *P < 0.05, **P < 0.01, ***P < 0.001, ▲P = 0.08 vs. Control; ns, not significant vs. no PPI. Bar = 100 μm” should read “DAPI was used for nuclear staining. *P < 0.05, **P < 0.01, ▲P = 0.08 vs. Control; ns, not significant vs. no PPI. Bar = 100 μm.” There are errors in the r and P values in Fig 3. The r value of the lower middle “no PPI” and “RANTES/GAPDH” graph should be “0.05”. The P value of the figure lower right “PPI” and “ANTES/GAPDH” should be “0.20”. The authors have provided the corrected versions of the figures.

**Fig 1:**
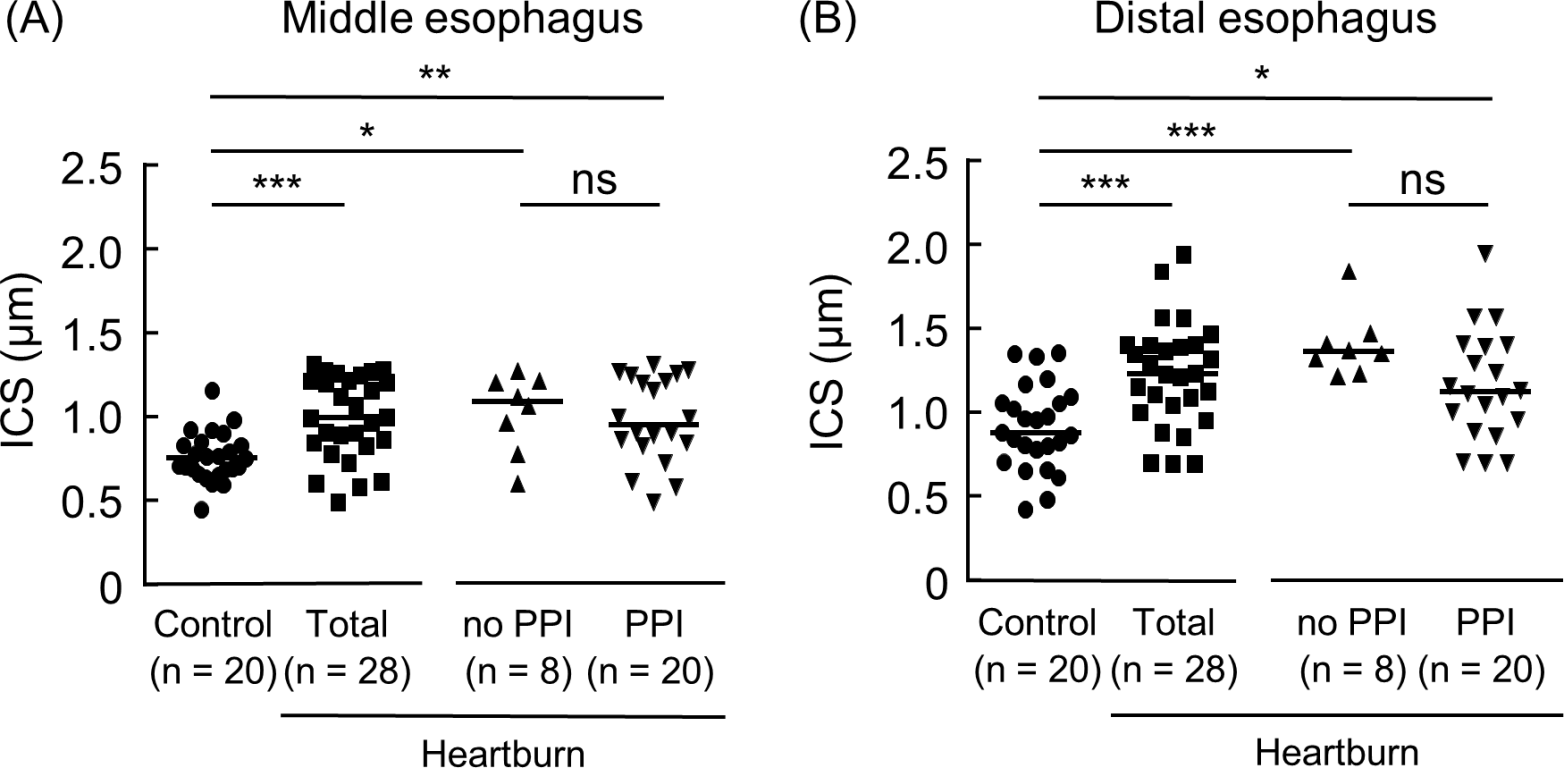
Intercellular space (ICS) diameters of the esophagus in patients with heartburn patients and controls. Middle and distal esophageal biopsy specimens were stained with hematoxylin and eosin. Intercellular space diameters of the middle (A) and distal (B) esophagus at the basal layers of squamous epithelial cells were then measured in control samples and heartburn patients (Total). The heartburn patients (Total) consist of the subjects who were not taking a PPI (no PPI) and who were (PPI). The horizontal line in each sample indicates the median value. *P < 0.05, **P < 0.01, ***P < 0.001 vs. Control; ns, not significant vs. no PPI.

**Fig 2:**
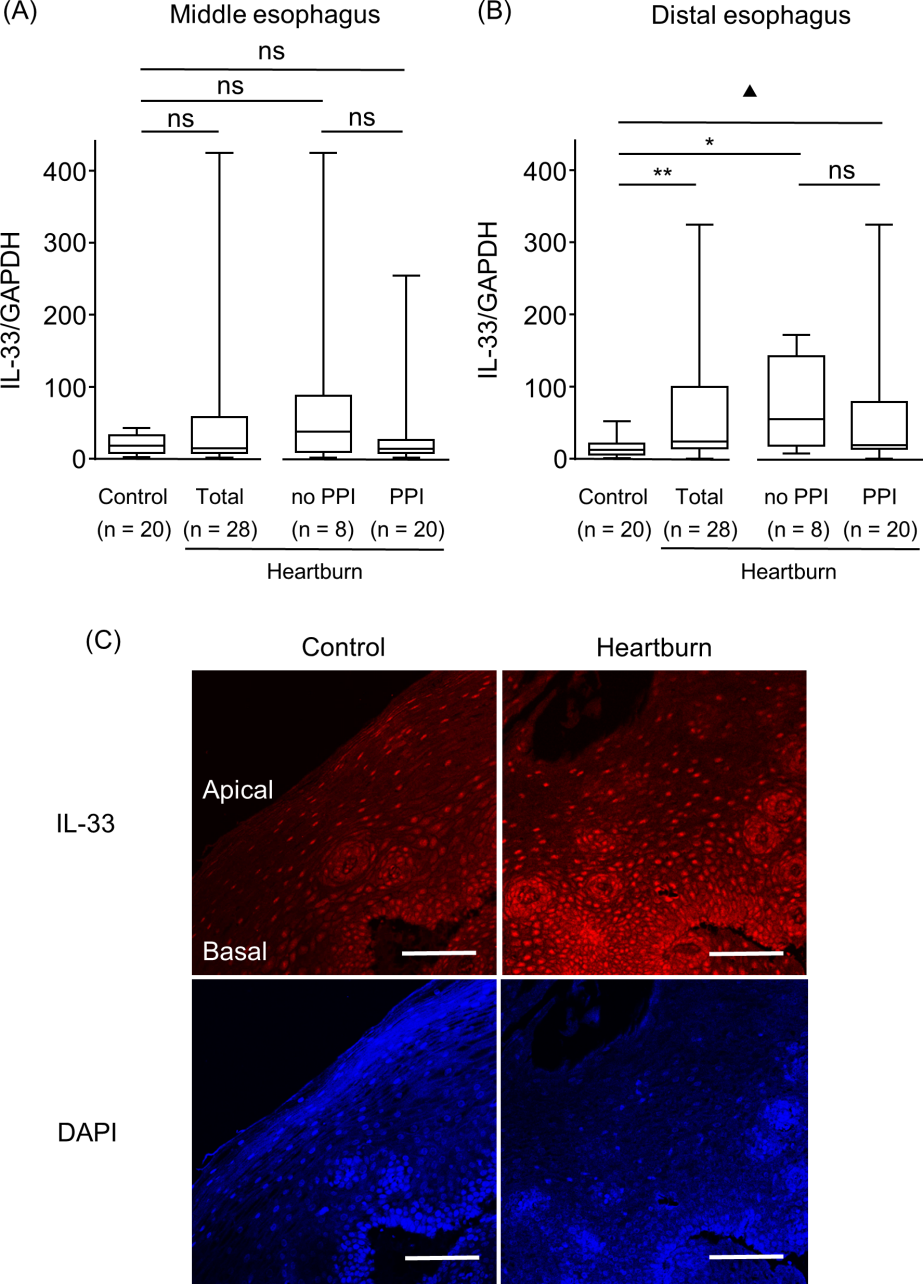
IL-33 expression in esophageal epithelial layers of patients with heartburn and controls. IL-33 mRNA expression was analyzed using qRT-PCR and was compared at the middle (A) and distal (B) esophagus of control samples, and the heartburn patients (Total), the subjects who were not taking a PPI (no PPI) and who were (PPI). (C) Representative immunofluorescence staining of IL-33 in the apical and basal layers of the distal esophagus of a control sample and a heartburn patient with taking a PPI is shown. DAPI was used for nuclear staining. *P < 0.05, **P < 0.01, ▲P = 0.08 vs. Control; ns, not significant vs. no PPI. Bar = 100 μm.

**Fig 3:**
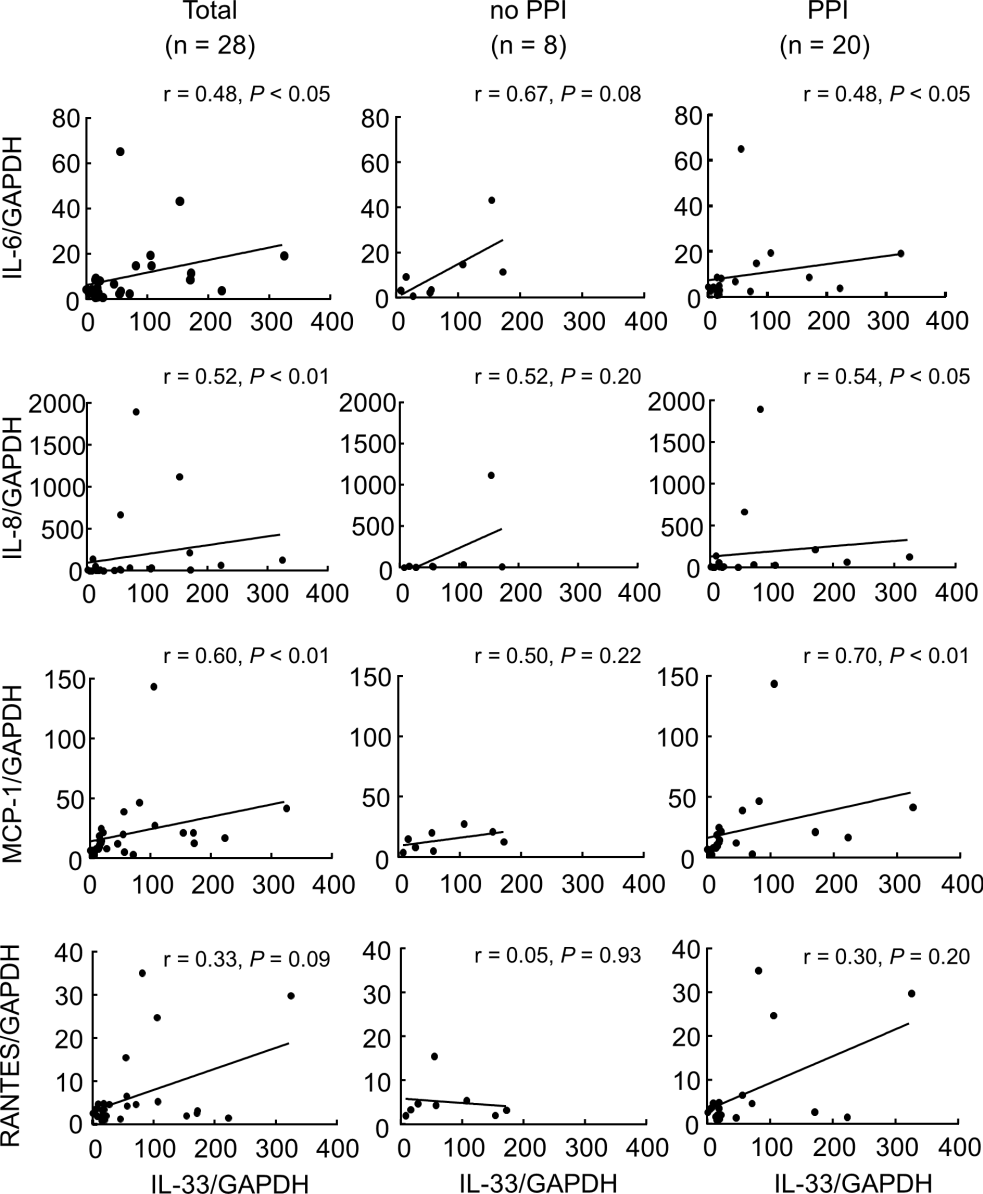
Analysis of the correlation of IL-33 and other inflammatory cytokines. Correlation of IL-33 mRNA levels and the mRNA levels of (A) IL-6, (B) IL-8, (C) MCP-1 and (D) RANTES in biopsy specimens from the distal esophagus of patients with heartburn, and patients who were not taking a PPI (no PPI) and who were (PPI) was performed by calculation of Spearman’s rank correlation coefficient.

## References

[1] Sei H, Oshima T, Shan J, Wu L, Yamasaki T, Okugawa T, et al (2016) Esophageal Epithelial-Derived IL-33 Is Upregulated in Patients with Heartburn. PLoS ONE 11(4): e0154234 doi:10.1371/journal.pone.0154234 27111066 10.1371/journal.pone.0154234 PMC4844101

